# Prevalence of headache and its interference in the activities of daily living in female adolescent students

**DOI:** 10.1590/0103-0582201432212113

**Published:** 2014-06

**Authors:** Alaine Souza Lima, Rodrigo Cappato de Araújo, Mayra Ruana de A. Gomes, Ludmila Remígio de Almeida, Gabriely Feitosa F. de Souza, Samara Barreto Cunha, Ana Carolina R. Pitangui

**Affiliations:** 1 UPE, Petrolina, PE, Brasil

**Keywords:** headache, adolescent, activities of daily living, pain, puberty

## Abstract

**OBJECTIVE:**

To describe the prevalence of headache and its interference in the activities of daily living (ADL) in female adolescent students.

**METHODS:**

This descriptive cross-sectional study enrolled 228 female adolescents from a public school in the city of Petrolina, Pernambuco, Northeast Brazil, aged ten to 19 years. A self-administered structured questionnaire about socio-demographic characteristics, occurrence of headache and its characteristics was employed. Headaches were classified according to the International Headache Society criteria. The chi-square test was used to verify possible associations, being significant *p*<0.05.

**RESULTS:**

After the exclusion of 24 questionnaires that did not met the inclusion criteria, 204 questionnaires were analyzed. The mean age of the adolescents was 14.0±1.4 years. The prevalence of headache was 87.7%. Of the adolescents with headache, 0.5% presented migraine without pure menstrual aura; 6.7%, migraine without aura related to menstruation; 1.6%, non-menstrual migraine without aura; 11.7%, tension-type headache and 79.3%, other headaches. Significant associations were found between pain intensity and the following variables: absenteeism (*p*=0.001); interference in ADL (*p*<0.001); medication use (*p*<0.001); age (*p*=0.045) and seek for medical care (*p*<0.022).

**CONCLUSIONS:**

The prevalence of headache in female adolescents observed in this study was high, with a negative impact in ADL and school attendance.

## Abstract (es)

**OBJETIVO:**

Determinar la prevalencia de cefalea y su interferencia en las actividades de vida diaria (AVD) en adolescentes escolares del sexo femenino.

**MÉTODOS:**

Estudio descriptivo transversal realizado con 228 adolescentes del sexo femenino matriculadas en una escuela pública del municipio de Petrolina, Pernambuco, con edades de 10 a 19 años. Se empleó un cuestionario estructurado autoaplicado con cuestiones sobre los datos sociodemográficos, ocurrencia de cefalea y sus características. Se clasificaron las cefaleas según los criterios de la Sociedad Internacional de Cefalea. Se utilizó la prueba del chi-cuadrado para verificar posibles asociaciones y se adoptó un nivel de significancia de *p*<0,05.

**RESULTADOS:**

Después de la exclusión de 24 cuestionarios debido a que no se rellenaban los criterios de inclusión, se analizaron 204 cuestionarios. El promedio de edad de las adolescentes fue de 14,0±1,4 años. La prevalencia de cefalea fue de 87,7%. De las adolescentes con cefalea, 0,5% presentaron migraña sin aura menstrual pura; 6,7%, migraña sin aura relacionada a la menstruación; 1,6%, migraña sin aura no relacionada a la menstruación; 11,7%, cefalea transicional y 79,3%, otras cefaleas. Se encontraron asociaciones significativas entre la intensidad del dolor y las variables: absentismo (*p*=0,001); interferencia en las AVD (*p*<0,001); uso de medicamentos (*p*<0,001); edad (*p*=0,0045) y busca por médico (*p*<0,022).

**CONCLUSIONES:**

La prevalencia de cefalea verificada en este estudio fue elevada en las adolescentes. Resulta claro el impacto negativo de este síntoma sobre las AVD y la vida escolar, habiendo necesidad de estudios futuros sobre el tema.

## Introduction

According to the World Health Organization (WHO), adolescence ranges from ages 10 to 19^(^ 1 ^)^, and puberty refers to the transitional period between childhood and adulthood. During this time, individuals undergo important physical and mental changes that result in their growth^(^ 1 ^)^. 

The first menstrual cycle is called menarche^(^ 2 ^)^ and usually happens between 12 and 13 years of age^(^ 3 ^)^. It is considered one of the most remarkable milestones in a woman's life^(^ 2 ^,^ 3 ^)^. The menstrual period is often accompanied by a range of symptoms, such as headache^(^ 4 ^)^, which is a painful and disabling condition that affects the general population. It is usually underdiagnosed and undertreated, even though it should be seen as a warning sign^(^ 5 ^)^. It is considered a common affection among children and adolescents^(^ 3 ^,^ 6 ^)^, and its prevalence in this population ranges from 9.7 to 78.2%^(^ 5 ^,^ 7 ^-^ 9 ^)^, although this variation may be due to methodological differences in the studies^(^ 6 ^,^ 10 ^)^. 

In primary headaches, the etiology cannot be determined by the usual clinical or laboratory tests^(^ 3 ^,^ 11 ^)^. Main examples are migraines and tension headaches^(^ 11 ^)^. Migraines are predominant in women and can range from moderate to severe, occasionally leading to functional disability^(^ 12 ^)^. Tension headaches are considered one of the most common forms of headache, although few studies have been conducted on them^(^ 12 ^)^. They have an incidence of 10% among women with menstrual headache^(^ 13 ^)^. 

An association between headaches and female sexual hormones levels may be observed. This is due to the fact that alterations in estradiol levels are determinant for some types of neurological disorders, such as migraines, since symptoms change according to the phases of the ovarian cycle^(^ 14 ^)^. This may explain why headaches are more prevalent in women than in men^(^ 15 ^)^. 

Headache is often associated with a significant drop in quality of life, negatively affecting school and work performance and activities of daily living (ADL)^(^ 16 ^)^. It is considered a cause of school absenteeism among children and adolescents^(^ 6 ^,^ 17 ^)^. In addition to these negative effects, headaches may have even more dramatic consequences for this population by triggering negative emotions such as sadness, anxiety, or anger^(^ 18 ^)^. 

Based on these data and considering the high prevalence and severity of symptoms, headaches currently present as a public health problem^(^ 19 ^)^. This highlights the negative impact of headaches on ADL for adolescents who suffer from this condition. Due to the higher prevalence among women, further research on this specific population is important. Therefore, this study aimed to determine the prevalence of headaches and its impact on ADL in female adolescents in a public school in Petrolina, state of Pernambuco, Brazil. 

## Method

This descriptive cross-sectional study was conducted with 288 female adolescents between 10 and 19 years old enrolled at a public school in Petrolina, Pernambuco, Brazil, between may and july 2012. School selection was based on the following criteria: large public school in an urban area (over 1000 students) offering both middle and high school classes. After the criteria had been defined, seven schools were eligible, and one of them was selected using a computer software. In order to familiarize students with the project, we spread information about it in the school. We handed out an informative letter and the informed consent form to the students' parents in order to inform them of the objectives and procedures of the study.

All participants and their carers were informed of the research procedures, in compliance with Resolution 196/96 of the Brazilian National Health Council. This study was approved by the Research Ethics Committee of the Universidade de Pernambuco under protocol no. 50/12.

The number of subjects was assessed using the WinPepi software. We considered the population of 584 female students enrolled at the school, an estimated proportion of adolescents with headache of 19.5%^(^ 7 ^)^, an absolute precision of 5%, and a loss of 20%, for a total of 171 adolescents. However, in order to have a better representation and distribution of the sample, 15 classes (60% of the total number) were randomly selected. Finally, we defined a minimum of 15 girls per class. The students in each class were assigned a number and were randomly selected according to inclusion criteria, which were: being appropriately enrolled at the school; being between 10 and 19 years old; and having returned the consent form signed and dated by their carer. Exclusion criteria were: having physical, behavioral and/or psychological alterations that would prevent them from filling out the data collection instrument; having neurological alterations; having facial trauma; using hormone medications, anticonvulsants and prophylactics for headache; and being pregnant or breastfeeding for the previous six months. 

**Figure 1:**
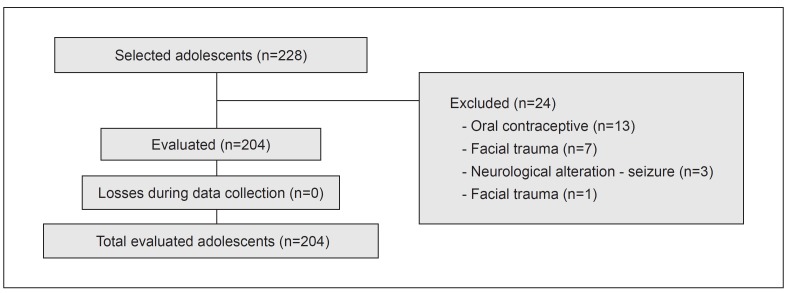
Eligibility diagram

From the overall sample, 13 girls were excluded for using oral contraceptives; seven for having facial trauma; one for having neurological alterations (seizure); one for having facial trauma and neurological alterations (seizure); one for taking anticonvulsants; and one for using progestogen-based medication. The final sample was composed of 204 adolescents. Sample eligibility criteria and data collection steps can be seen in Figure 1.

We used a structured questionnaire formulated by us with questions on sociodemographic data, occurrence of headache and its characteristics. Before data collection, a pilot study was conducted with ten adolescents in order to improve the questionnaire, which was revised for ease of interpretation. Headaches were classified according to the criteria of the International Headache Society (SIC)^(^ 12 ^)^. The following aspects were evaluated: intensity of pain, measured by the Visual Numeric Scale (VNS) and classified as mild, moderate, or severe; pain duration; impact on ADLs, divided into "yes" or "no" and classified as mild, significant, or disabling; pain characteristics, classified as throbbing or not; pain location, classified as unilateral or bilateral; and associated symptoms, such as photophobia, phonophobia, nausea, and vomiting. The questionnaire was self-administered, and a trained researcher supervised the adolescents to answer any possible questions.

Statistical analysis was performed using the *Statistical Package for the Social Sciences* (SPSS) software version 20.0. The confidence interval (95%CI) was calculated using the WinPepi software. Continuous variables were expressed as measures of central tendency and dispersion, and categorical variables were expressed as absolute and relative frequencies. The chi-square test was used to assess the associations. All analyses used a significance level of *p*<0.05.

## Results

We analyzed 204 questionnaires. Mean age was 14.0±1.4 years, and prevalence of headache was 87.7% (n=179; 95%CI 82.4-91.9). Table 1 shows the absolute and relative values of headache classifications, as indicated by data collected from the questionnaires. 

**Table 1:**
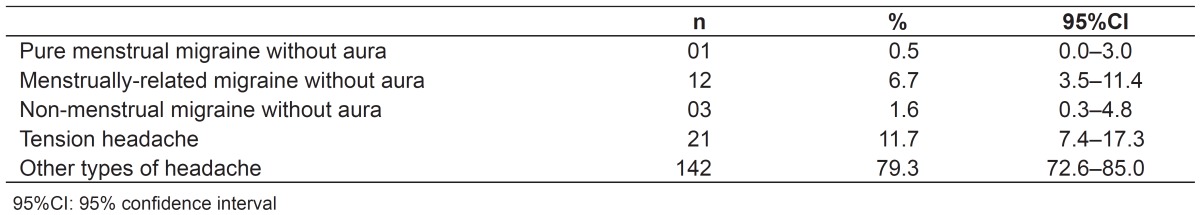
Classification of headaches (n=179)

Regarding intensity of pain, headache was mild in 13.4% (n=24) of the adolescents, moderate in 69.2% (n=124), and severe in 17.3% (n=31). For 84.9% (n=152) of the participants, headache had an impact on ADL. Out of these, 46.0% (n=70) reported that pain had a significant or disabling impact on ADL. 

Of the total number of adolescents with headache, 61.4% (n=110) reported at least one associated symptom. The most prevalent ones were photophobia (60.9%, n=67), phonophobia (56.3%, n=62), and nausea (22.7%, n=25).

Need for pain medication was reported by 70.3% (n=126) of the subjects. However, only 26.2% (n=47) of them reported seeking medical attention due to pain complaints. School absenteeism caused by headache was reported by 31.8% (n=57) of the students.

There was a statistically significant association between intensity of pain and the following variables: school absenteeism, impact on ADL, medical care seeking, and use of medication (Table 2).

**Table 2:**
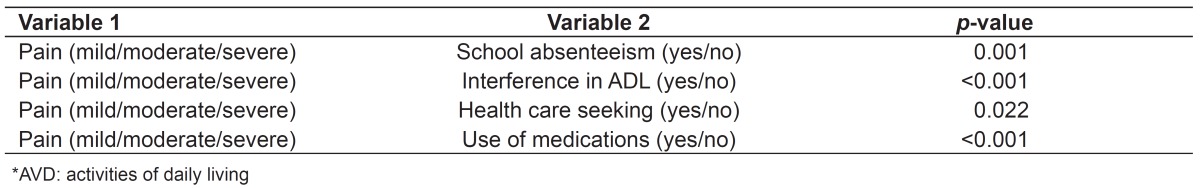
Associations between intensity of pain, school absenteeism, impact on activities of daily living, health care seeking and need for medications (n=179)

## Discussion

This study aimed to determine the prevalence of headache and its effects on ADL in female adolescent students. Headache is a common and disabling health problem that affects people worldwide^(^ 5 ^,^ 20 ^)^, with impacts on health costs^(^ 8 ^)^. High prevalences were found by some authors, such as Bahrami et al^(^ 5 ^)^, who reported a prevalence of 78.2%. Prevalence of headache among children and adolescents is highly variable in the literature, ranging from 9.7 to 78.2%^(^ 5 ^,^ 7 ^-^ 9 ^)^. This variation may be explained by differences in methods, diagnostic criteria^(^ 21 ^)^, and geographic location^(^ 22 ^)^.

A high prevalence of headache (87.8%) was observed among the adolescents in this study. Many authors noted that women are significantly more affected by this condition than men^(^ 5 ^,^ 23 ^,^ 24 ^)^. This is thought to be caused by the various alterations in the brain brought on by the effects of estrogen on the nervous system^(^ 14 ^)^. The frequency of some neurological disorders, such as migraine, may increase when estradiol levels change^(^ 14 ^)^. However, the relationship between menstrual period and tension headache remains unclear^(^ 13 ^)^.

It bears stressing that adolescents who used hormone medications, such as oral contraceptives, were excluded from this study. The use of oral contraceptives may have deleterious effects on more sensitive women. This leads to worsening of migraines, particularly during the hormone withdrawal phase^(^ 25 ^)^. In a study with Taiwanese adolescent students, intensity of pain was found to be associated with occurrence of menarche^(^ 26 ^)^. Another factor that should be considered is family history, since headache frequency is related to the frequency of symptoms in the adolescents' mothers^(^ 27 ^)^. 

This study showed a statistically significant association between intensity of pain and the following variables: school absenteeism, impact on ADL, medical care seeking, and use of medications. However, these results were expected. The more intense the pain, the greater the impact on routine activities and, consequently, the greater the need for medical attention. 

Use of medication to alleviate the pain was reported by most adolescents (70.4%). As other authors have observed^(^ 23 ^)^, most of the subjects in this population self-medicated, since the proportion of adolescents who sought medical care for this complaint was very low (26.3%). However, in addition to family history, overuse of headache medication is believed to worsen headaches^(^ 28 ^)^. Thus, it is important to have health policies to help inform the population of the consequences of the frequent and non-prescribed use of headache medications.

Headache is a concern when present in children and adolescents due to its negative impact on school attendance and performance, as well as on family and interpersonal relationships, compromising quality of life in this population^(^ 9 ^,^ 29 ^)^. In this study, headache was a cause of school absenteeism for 31.8% of the adolescents. In addition, headache is considered to interfere in ADL^(^ 24 ^)^. This was confirmed in this study by 84.9% of the students, with most of them reporting a significant or disabling impact on ADL. Considering the negative repercussions in the life of adolescents, it is important to study the effects of headache on ADL. In spite of that, headache is usually underdiagnosed and undertreated^(^ 5 ^,^ 29 ^)^, and new therapeutic approaches are necessary to reduce pain and increase quality of life^(^ 29 ^)^.

However, this study has some limitations which should be acknowledged, particularly the use of a self-administered questionnaire for the diagnosis of headache, which could generate a memory bias. This limitation is inherent to cross-sectional retrospective studies^(^ 30 ^)^. In spite of that, many authors consider questionnaires to be appropriate for epidemiological studies due to the fact that the diagnosis of headache is based on the presence of specific characteristics and symptoms^(^ 8 ^)^. 

Another limitation was that the sample was specific to a public school in a small town in Brazil, meaning the findings cannot be extended to other populations. In addition, family history was not analyzed. This opens the way for new studies that consider the mentioned limitations and compare the presence and intensity of headaches between adolescents who take and do not take oral contraceptives.

Based on the data presented in this study, we conclude that prevalence of headache was high among the adolescents. There was a significant association between intensity of pain and the following variables: school absenteeism, impact on ADL, medical care seeking, and use of medications. The negative impact of headache symptoms on ADL and school performance was clear. A high rate of self-medication and a low rate of health care seeking were also observed in the adolescents, possibly due to a misconception that headache is a common problem that doesn't require medical attention. In this context, and considering the negative repercussions of headache in the life of the adolescents, there is a need for future studies on this condition in order to develop efficient preventive and therapeutic measures to alleviate the symptoms.
