# Supplementary material for: Prevalence of headache and its interference in the activities of daily living in female adolescent students
Source: Rev Paul Pediatr. 2014 Jun;32(2):256–61. doi: 10.1590/0103-0582201432212113 (PMC4183010; doi:10.1590/0103-0582201432212113)
Supplement: Translation

# Translation: Prevalência de cefaleia e sua interferência nas atividades de vida diária em adolescentes escolares do sexo feminino

**Authors:** Alaine Souza Lima, Rodrigo Cappato de Araújo, Mayra Ruana de A. Gomes, Ludmila Remígio de Almeida, Gabriely Feitosa F. de Souza, Samara Barreto Cunha, Ana Carolina R. Pitangui

**Affiliations:** 1 UPE, Petrolina, PE, Brasil

**Keywords:** cefaleia, adolescente, atividades cotidianas, dor, puberdade

## Abstract

**OBJETIVO:**

Determinar a prevalência de cefaleia e sua interferência nas atividades de vida diária (AVD) em adolescentes escolares do sexo feminino.

**MÉTODOS:**

Estudo descritivo transversal realizado com 228 adolescentes do sexo feminino matriculadas em uma escola pública do município de Petrolina, PE, com idades de dez a 19 anos. Empregou-se um questionário estruturado autoaplicado com questões acerca dos dados sociodemográficos, ocorrência de cefaleia e suas características. Classificaram-se as cefaleias segundo os critérios da Sociedade Internacional de Cefaleia. Utilizou-se o teste do qui-quadrado para verificar possíveis associações e adotou-se um nível de significância de *p*<0,05.

**RESULTADOS:**

Após a exclusão de 24 questionários devido ao não preenchimento dos critérios de inclusão, analisaram-se 204 questionários. A idade média das adolescentes foi de 14,0±1,4 anos. A prevalência de cefaleia foi de 87,7%. Das adolescentes com cefaleia, 0,5% apresentaram migrânea sem aura menstrual pura; 6,7%, migrânea sem aura relacionada à menstruação; 1,6%, migrânea sem aura não relacionada à menstruação; 11,7%, cefaleia tensional e 79,3%, outras cefaleias. Encontraram-se associações significativas entre intensidade da dor e as variáveis: absenteísmo (*p*=0,001); interferência nas AVD (*p*<0,001); uso de medicamentos (*p*<0,001); idade (*p*=0,045) e procura médica (*p*<0,022).

**CONCLUSÕES:**

A prevalência de cefaleia verificada neste estudo foi elevada nas adolescentes, com impacto negativo sobre as AVD e a vida escolar.

## Introdução

Segundo a Organização Mundial da Saúde (OMS), a adolescência compreende as idades de dez e 19 anos^(^ ^1^ ^)^, sendo a puberdade correspondente ao período de transição entre a infância e a vida adulta, no qual ocorrem importantes transformações físicas e mentais que resultam no amadurecimento do indivíduo^(^ ^1^ ^)^. 

O início da menstruação é denominado menarca^(^ ^2^ ^)^ e geralmente ocorre entre os 12 e 13 anos de idade^(^ ^3^ ^)^, sendo considerado um dos marcos mais expressivos na vida de uma mulher^(^ ^2^ ^,^ ^3^ ^)^. O período menstrual comumente é acompanhado por uma variedade de sintomas, tal como a cefaleia^(^ ^4^ ^)^, condição dolorosa e incapacitante que acomete a população em geral, porém muitas vezes é subdiagnosticada e subtratada, devendo, no entanto, ser vista como um sinal de alerta^(^ ^5^ ^)^. É considerada uma afecção comum em crianças e adolescentes^(^ ^3^ ^,^ ^6^ ^)^ e sua prevalência nessa população varia de 9,7 a 78,2%^(^ ^5^ ^,^ ^7^ ^-^ ^9^ ^)^; contudo, acredita-se que essa variedade nas taxas de prevalência possa ser decorrente das diferenças metodológicas dos estudos^(^ ^6^ ^,^ ^10^ ^)^. 

As cefaleias primárias são aquelas que não apresentam etiologia demonstrável pelos exames clínicos ou laboratoriais habituais^(^ ^3^ ^,^ ^11^ ^)^ e têm como principais exemplos a migrânea e a cefaleia do tipo tensional^(^ ^11^ ^)^. A migrânea predomina no sexo feminino e apresenta intensidade moderada a grave, podendo levar à incapacidade funcional^(^ ^12^ ^)^. Já a cefaleia do tipo tensional é considerada uma das formas mais comuns de dor cefálica, embora ainda seja pouco estudada^(^ ^12^ ^)^, sendo sua incidência de cerca de 10%, em mulheres com cefaleia menstrual^(^ ^13^ ^)^. 

A associação entre as cefaleias e os níveis de hormônios sexuais femininos pode ser observada em decorrência das modificações dos níveis de estradiol serem determinantes para alguns distúrbios neurológicos, tal como a migrânea, já que ocorrem alterações de sintomas durante as diferentes fases do ciclo ovariano^(^ ^14^ ^)^. Esse fato pode justificar a maior prevalência de cefaleias em mulheres, comparadas aos homens^(^ ^15^ ^)^. 

Observa-se frequentemente diminuição significativa na qualidade de vida associada à presença das cefaleias, prejudicando, assim, o desempenho na escola, no trabalho e durante a realização das atividades de vida diária (AVD)^(^ ^16^ ^)^, sendo ainda considerada causa de absenteísmo escolar em crianças e adolescentes^(^ ^6^ ^,^ ^17^ ^)^. Além dessas repercussões negativas, as cefaleias podem desencadear o surgimento de emoções negativas como tristeza, ansiedade ou raiva^(^ ^18^ ^)^. 

Diante do exposto e devido à alta prevalência e à gravidade do quadro sintomatológico, constata-se que as cefaleias apresentam-se atualmente como um problema de Saúde Pública^(^ ^19^ ^)^, com impacto negativo nas atividades cotidianas dos adolescentes. Sendo assim, este estudo teve como objetivo determinar a prevalência de cefaleia e sua interferência nas AVD em adolescentes escolares do sexo feminino de uma escola pública do município de Petrolina, PE. 

## Método

Estudo transversal, realizado com 228 adolescentes do sexo feminino de dez a 19 anos, matriculadas em uma escola pública no município de Petrolina, PE, Brasil, no período de maio a julho de 2012. A escolha da escola se deu pelos seguintes critérios: escola estadual localizada em zona urbana, de grande porte (acima de mil alunos) e que apresentasse turmas do ensino fundamental e médio. Depois dos critérios estabelecidos, sete escolas foram elegíveis. Em seguida, selecionou-se uma escola a ser avaliada por sorteio por meio de um programa de computador. Para apresentação e familiarização com o projeto, realizou-se a divulgação na escola, sendo entregue uma carta explicativa e o termo de consentimento livre e esclarecido aos responsáveis das alunas para o conhecimento dos objetivos da pesquisa e da rotina do experimento.

Todas as participantes e seus respectivos responsáveis foram informados sobre os procedimentos da pesquisa, segundo a resolução nº 196/96 do Conselho Nacional da Saúde. Este estudo foi aprovado pelo Comitê de Ética em Pesquisa da Universidade de Pernambuco.

Para quantificar o número de sujeitos, utilizou-se o programa WinPepi. Considerou-se a população de 584 estudantes do sexo feminino matriculadas na escola, proporção estimada de adolescentes com cefaleia de 19,5%^(^ ^7^ ^)^, precisão absoluta de 5% e perda de 20%, totalizando 171 adolescentes. No entanto, para que houvesse uma melhor representação e distribuição da amostra, selecionaram-se 15 turmas, por meio de sorteio, o que representou 60% do total de turmas da escola. Por fim, definiu-se um número mínimo de 15 meninas por turma. As alunas de cada turma foram codificadas numericamente e, posteriormente, realizou-se a randomização das participantes, respeitando-se os critérios de inclusão do estudo. 

Incluíram-se as adolescentes que atenderam aos seguintes critérios: estar devidamente matriculada na escola; idade de dez a 19 anos e ter entregado o termo de consentimento devidamente datado e assinado pelo responsável. Os critérios de exclusão foram: apresentar alterações no estado físico, comportamental e/ou psicológico que impossibilitassem o preenchimento dos instrumentos de coleta; alteração neurológica; trauma de face; uso de medicamentos hormonais, anticonvulsivantes e profiláticos para tratamento de cefaleia e estar grávida ou amamentando nos últimos seis meses. 

Do total da amostra, excluíram-se 13 voluntárias por uso de anticoncepcional oral, sete por terem sofrido trauma de face, uma por apresentar alteração neurológica (convulsão), uma por ter sofrido trauma de face e apresentar alteração neurológica (convulsão), uma por uso de medicamento anticonvulsivante e uma por uso de medicamento à base de hormônios progestágenos, sendo, portanto, o estudo composto por 204 adolescentes. A elegibilidade da amostra e as etapas da coleta podem ser visualizadas na Figura 1. 

**Figura 1:** Diagrama de elegibilidade das adolescentes

Aplicou-se um questionário estruturado elaborado pelos pesquisadores, que constava de questões acerca dos dados sociodemográficos, ocorrência de cefaleia e suas características. Antes da coleta de dados, realizou-se um estudo-piloto com dez adolescentes para aprimorar o questionário, modificando-o de modo facilitar a interpretação. Classificaram-se as cefaleias segundo os critérios diagnósticos propostos pela Sociedade Internacional de Cefaleia (SIC)^(^ ^12^ ^)^. Avaliaram-se os seguintes quesitos: intensidade da dor, mensurada pela Escala Visual Numérica (EVN) e categorizada como leve, moderada e grave; duração da dor; interferência nas AVD, considerada pelos descritores sim e não e classificada em pouco, significante e incapacitante; característica da dor, qualificada como latejante ou não; localização da dor, caracterizada em unilateral ou bilateral; e sintomas associados à cefaleia, como fotofobia, fonofobia, náuseas e vômitos. O questionário foi autoaplicado e, durante o preenchimento, uma pesquisadora treinada supervisionou as adolescentes para solucionar possíveis dúvidas.

Para análise estatística, os dados foram processados e analisados com o programa *Statistical Package for the Social Sciences* (SPSS), versão 20.0. Para o cálculo do intervalo de confiança de 95% (IC95%), utilizou-se o programa WinPepi. As variáveis contínuas foram apresentadas sob a forma de medidas de tendência central e dispersão e as variáveis categóricas, em frequências absolutas e relativas. Para associação, utilizou-se o teste do qui-quadrado. Em todas as análises, adotou-se nível de significância de *p*<0,05.

## Resultados

Analisaram-se 204 questionários de adolescentes com idade média de 14,0±1,4 anos, verificando-se prevalência de cefaleia de 87,7% (n=179; IC95% 82,4-91,9). Na Tabela 1, podem ser visualizados os valores absolutos e relativos referentes à classificação das cefaleias, a partir das informações colhidas nos questionários.

**Tabela 1:** Classificação das cefaleias (n=179)

Quanto à intensidade da dor, 13,4% (n=24) das adolescentes relataram a cefaleia como leve, 69,2% (n=124) como moderada e 17,3% (n=31) como grave. A cefaleia foi considerada um fator de interferência nas AVD por 84,9% (n=152) das adolescentes, sendo que, destas, 46,0% (n=70) relataram que a dor interferia significativamente ou eram incapazes de desempenhar as AVD. 

Do total das adolescentes com cefaleia, 61,4% (n=110) relataram presença de pelo menos um sintoma associado, sendo os mais prevalentes a fotofobia, com 60,9% (n=67), a fonofobia, com 56,3% (n=62) e as náuseas, com 22,7% (n=25).

A necessidade de medicamento para alívio da dor foi relatada por 70,3% (n=126) das participantes. No entanto, apenas 26,2% (n=47) das adolescentes referiram procurar ajuda médica devido à queixa álgica. Absenteísmo escolar foi referido por 31,8% (n=57) das escolares em decorrência da cefaleia.

 Associações estatisticamente significativas foram encontradas entre intensidade da dor e as variáveis absenteísmo escolar, interferência nas AVD, procura médica e uso de medicamentos (Tabela 2).

**Tabela 2:** Associações entre intensidade da dor, absenteísmo escolar, interferência nas atividades de vida diária, procura médica e necessidade de medicamentos (n=179)

## Discussão

O objetivo do presente estudo foi determinar a prevalência de cefaleia e sua interferência nas AVD em adolescentes escolares do sexo feminino. A cefaleia é um problema de saúde frequente e incapacitante que acomete indivíduos em todo o mundo^(^ ^5^ ^,^ ^20^ ^)^, com impacto quanto aos custos em saúde^(^ ^8^ ^)^. Altas prevalências foram encontradas por alguns autores, como Bahrami *et al**^(^* ^5^ ^)^, que verificaram prevalência de 78,2% em seu estudo. Na literatura, a prevalência de cefaleia em crianças e adolescentes é muito variável, apresentando-se de 9,7 a 78,2%^(^ ^5^ ^,^ ^7^ ^-^ ^9^ ^)^. Tal diversidade encontrada nos estudos provavelmente pode ser justificada pelas diferenças metodológicas adotadas, pelos critérios diagnósticos utilizados^(^ ^21^ ^)^ e pela localização geográfica^(^ ^22^ ^)^.

Verificou-se elevada prevalência de cefaleia nas adolescentes deste estudo, com taxas de 87,8%. Vários autores observaram que as mulheres, comparadas aos homens, são significativamente mais afetadas por tal sintoma^(^ ^5^ ^,^ ^23^ ^,^ ^24^ ^)^. Postula-se que a maior prevalência de cefaleias em indivíduos do sexo feminino possa ser explicada pelas diversas alterações que ocorrem no cérebro, devido aos efeitos que o estrogênio ocasiona no sistema nervoso^(^ ^14^ ^)^. Distúrbios neurológicos, como a migrânea, podem aumentar em frequência quando os níveis de estradiol se alteram^(^ ^14^ ^)^. Contudo, a relação entre o período menstrual e a cefaleia do tipo tensional ainda não é clara^(^ ^13^ ^)^.

Vale ressaltar que, neste estudo, excluíram-se as adolescentes que utilizavam medicamentos hormonais, como os anticoncepcionais orais. O uso de contraceptivos hormonais pode ter efeito maléfico em mulheres mais sensíveis, piorando o quadro da migrânea, particularmente durante o intervalo de retirada dos hormônios^(^ ^25^ ^)^. Em adolescentes escolares de Taiwan, verificou-se que a intensidade das dores de cabeça nos indivíduos do sexo feminino tem possível relação com a ocorrência da menarca^(^ ^26^ ^)^. Outro fator a ser considerado é o histórico familiar, visto que a frequência de cefaleia relaciona-se à frequência de dores de cabeça da mãe^(^ ^27^ ^)^. 

Na presente pesquisa, houve associações estatísticas entre a intensidade da dor e as variáveis absenteísmo escolar, interferência nas AVD, procura médica e uso de medicamentos. No entanto, tais resultados eram esperados, pois entende-se que, quanto mais intensa a cefaleia, maior será o prejuízo na realização das atividades rotineiras e, consequentemente, maior será a necessidade de procurar ajuda médica. 

O uso de fármacos para o alívio da dor foi referido pela maioria das adolescentes (70,4%). Como verificado por outros autores^(^ ^23^ ^)^, pode-se inferir que boa parte dessa população realizava automedicação, pois a proporção de adolescentes que procuraram auxílio médico devido a essa queixa foi muito baixa (26,3%). No entanto, acredita-se que o uso abusivo de medicamentos para aliviar a dor de cabeça, além do histórico familiar, pode contribuir para a cronificação da cefaleia^(^ ^28^ ^)^. Nesse sentido, tornam-se fundamentais as políticas de saúde que busquem esclarecer a população quanto às consequências do uso rotineiro e sem prescrição de fármacos para aliviar as dores de cabeça.

A cefaleia é motivo de preocupação em crianças e adolescentes devido ao impacto negativo exercido na assiduidade e no desempenho escolar, bem como nas relações familiares e interpessoais, prejudicando, assim, a qualidade de vida dessa população^(^ ^9^ ^,^ ^29^ ^)^. Neste estudo, a cefaleia foi razão de absenteísmo escolar em 31,8% das adolescentes analisadas. Além disso, é considerada como fator de interferência nas AVD^(^ ^24^ ^)^, situação também constatada neste estudo em 84,9% das escolares, sendo que a maioria relatou que esse problema interferia significativamente ou incapacitava a realização das AVD. Em decorrência das repercussões negativas na vida das adolescentes, tais como o declínio na realização das atividades acadêmicas e cotidianas, a influência da cefaleia no desempenho das AVD é um item fundamental a ser abordado. Apesar disso, constata-se que comumente a cefaleia é subdiagnosticada e subtratada^(^ ^5^ ^,^ ^29^ ^)^, sendo necessário desenvolver abordagens terapêuticas para reduzir a dor e melhorar a qualidade de vida^(^ ^29^ ^)^.

Contudo, algumas limitações do presente estudo devem ser relatadas, destacando-se o fato de ter sido utilizado um questionário autoaplicado para o diagnóstico das cefaleias, fato que poderia ocasionar um viés de memória, fraqueza esta inerente a estudos transversais retrospectivos^(^ ^30^ ^)^. Entretanto, diversos autores consideram que a utilização de questionários é adequada para estudos epidemiológicos pelo fato de o diagnóstico das cefaleias ser baseado na presença de sintomas e de características específicas^(^ ^8^ ^)^. 

Outra limitação foi o fato de a amostra deste estudo ter sido específica de uma escola pública de uma cidade do interior do Brasil; dessa forma, os resultados encontrados não podem ser generalizados para outras populações. Além disso, não se analisaram os antecedentes familiares de cefaleia e, assim, sugere-se a realização de novos estudos que considerem as limitações citadas e que comparem a presença e a intensidade da cefaleia em adolescentes que fazem uso rotineiro ou não de anticoncepcionais.

Em suma, conclui-se com os dados do presente estudo que a prevalência de cefaleia foi elevada nas adolescentes analisadas. Observaram-se associações significativas entre a intensidade da dor e as variáveis absenteísmo escolar, interferência nas AVD, procura médica e uso de medicamento, ficando claro o impacto negativo que essa sintomatologia pode causar nas AVD e no meio escolar. Notou-se ainda elevada taxa de automedicação pelas adolescentes e baixo percentual de procura médica, talvez por considerarem a cefaleia um problema comum e que não requer maior atenção. Nesse contexto, em decorrência das repercussões negativas que a cefaleia pode ocasionar na vida das adolescentes, destaca-se a necessidade de estudos futuros acerca do tema para se realizarem medidas preventivas e terapêuticas eficientes para o alívio dos sintomas.
